# One step fabrication of Silicon nanocones with wide-angle enhanced light absorption

**DOI:** 10.1038/s41598-018-22100-7

**Published:** 2018-03-05

**Authors:** Sara Magdi, Joumana El-Rifai, Mohamed A. Swillam

**Affiliations:** 1 Nanotechnology Program, American University in Cairo, AUC Avenue New Cairo, 11835 Cairo, Egypt; 2 Department of Physics, American University in Cairo, AUC Avenue New Cairo, 11835 Cairo, Egypt

## Abstract

We report the fabrication of an array of random Silicon nanocones using a KrF excimer laser. A 370 nm thick amorphous Silicon layer deposited on a glass substrate was used in the process. The fabricated nanocones showed a large and broadband absorption enhancement over the entire visible wavelength range. An enhancement up to 350% is measured at λ = 650 nm. Additionally, the laser irradiation caused the nanocones to crystallize. The effect of changing the laser parameters (i.e. energy density, time, and frequency) on the morphology and the absorption is studied and compared. Wide-angle anti-reflective properties have been observed for the fabricated nanocones with less than 10% reflection for angles up to 60°. The major limitation of amorphous silicon thin film solar cells is the reduced absorption. This problem could be solved if light is trapped efficiently inside the thin film without the need of increasing the film thickness. The random array of nanocones presented in this work showed a substantial increase in absorption over a wide angle, were fabricated at a low cost and are easily scalable. This technique offers a fast approach which could significantly help in overcoming the absorption limitation.

## Introduction

Thin film amorphous Silicon (a-Si) solar cells have gained an increased attention due to their low energy and cost requirements compared to their crystalline counterparts^1^. Thus, they offer an affordable alternative with a shorter energy-payback time^2^. However, the amorphous material suffers from a low carrier diffusion length, due to the randomness of its crystal structure, which significantly limits its thickness to a few hundred nanometers^3^. This small thickness reduces the absorption within the solar cell. Several techniques have been developed to overcome this limitation, such as surface texturing^3–8^, anti-reflection coatings^9^, plasmonics^10–12^, and photonic crystals^13^. One way to efficiently trap light inside the silicon active layer is to introduce a surface texture that could scatter light inside the Silicon under layer. In addition, surface texturing significantly reduces the reflection at a wide angle, solving the bad bare silicon reflection properties^14–22^.

Surface nanotexturing have been introduced on a-Si solar cells using several techniques. Usually the underlying substrate is textured using a chemical process where the texture propagates through the a-Si layer deposited on top^2–4,23,24^. Among various 3D nanostructures, silicon nanocones (NCs) are considered as the most efficient light trapping configuration for solar cells because of their improved refractive index matching with air^25^. Arrays of a-Si NCs have been fabricated using reactive ion etching along with a nanospheres template^26–28^. Although the process enhances the absorption in a-Si, this technique is expensive and requires multiple fabrication steps.

Excimer laser crystallization and formation of surface texturing has been previously reported^29–34^. The formation of a single conical nano-tip is reported upon single pulse laser irradiation of crystalline silicon^35–37^. In addition, surface roughness is observed upon amorphous and polycrystalline silicon irradiation with multiple pulses from an excimer laser^38–43^. Enhanced absorption is measured for some of these excimer laser induced surface texturing^39,44,45^. A very high frequency, up to 2000 Hz, is used to induce the surface roughness in some cases^44,45^. This extremely high frequency requires high amounts of energy which makes their fabrication not highly efficient for solar cells applications. Moreover, the formation of a well-defined array of nanocones with controllable dimensions, enhanced absorption and wide-angle anti-reflective properties has not yet been achieved. The effect of changing each laser parameter, such as laser exposure time and frequency, on the morphology and optical properties of the obtained structures were not studied.

Here, we report a lithography-free one step fabrication method of large area a-Si NCs using a KrF excimer laser. In this method, the silicon is not etched to produce the NCs as is customary with conventional methods. However, the silicon mass is re-distributed within the sample forming the NCs. Furthermore, this method is fast and does not require the use of a catalyst, special gases, vacuum, or clean room environment. Large and broadband absorption enhancement is obtained for nanostructured a-Si compared to the bare a-Si. Angle dependence analysis showed that the reduced reflection is stable for many angle of incidences. The current work provides a detailed systematic study on the effect of changing excimer laser parameters on the morphology and optical properties of a-Si.

## Results and Discussions

### Synthesis and characterization

**Figure 1:**
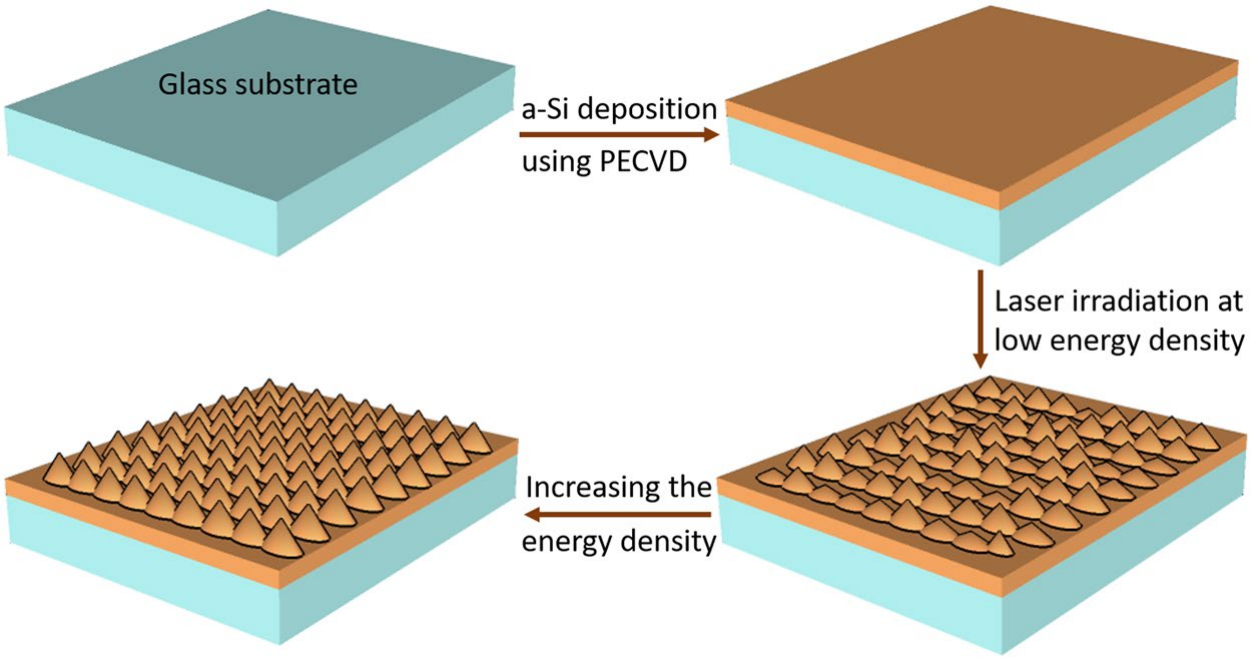
Sketch of the fabrication process of Silicon nanocones.

A 370 nm thick a-Si layer was deposited on glass slide substrates from a SiH_4_ source using plasma enhanced chemical vapor deposition PECVD (Oxford instruments). A none absorbing (i.e. transmitting) material such as glass was chosen as a substrate in order not to influence the absorption and reflection measurements of the silicon. Deposition parameters were fixed for all deposited a-Si (pressure = 1.5 Torr, table temperature = 250 °C, and power = 6 W). He gas was added to obtain uniform films with the ratio of SiH_4_:He set to 1:1^46,47^. Prior to deposition, glass slides were cleaned using acetone, isopropyl alcohol (IPA) and piranha solution. Cleaning and deposition were performed in a clean room environment. Figure 1 illustrates the whole fabrication process of Silicon nanocones using excimer laser.

The deposited a-Si films were then irradiated by a KrF excimer laser (Lambda Physik, Compex205, *λ* = 248 nm, pulse duration = 24 ns). The energy density, time and frequency of the laser pulse were varied throughout the experiments to analyze the effect of each parameter on the obtained NCs shape and their optical properties. The samples were then characterized using field emission scanning electron microscopy (FESEM) and atomic force microscopy (AFM) for morphology analysis. All cross section images are taken with 20° tilt. The absorption and reflection were measured using a spectrophotometer.

The Si nanocones (NCs) are formed by gradually increasing the laser energy density during laser exposure time. The energy density was gradually increased from 45 mJ/cm^2^ to a maximum energy in 40 seconds. The maximum energy density (E_max_) was varied between 215 mJ/cm^2^ to 520 mJ/cm^2^. The frequency (f) was initially fixed to 10 Hz and exposure time (T) was fixed to 40 seconds. It should be noted here that this energy is measured on the sample stage in air and the actual experiment is done in reduced pressure (3.5 mbar).

### Nanocones morphological and optical properties

**Figure 2:**
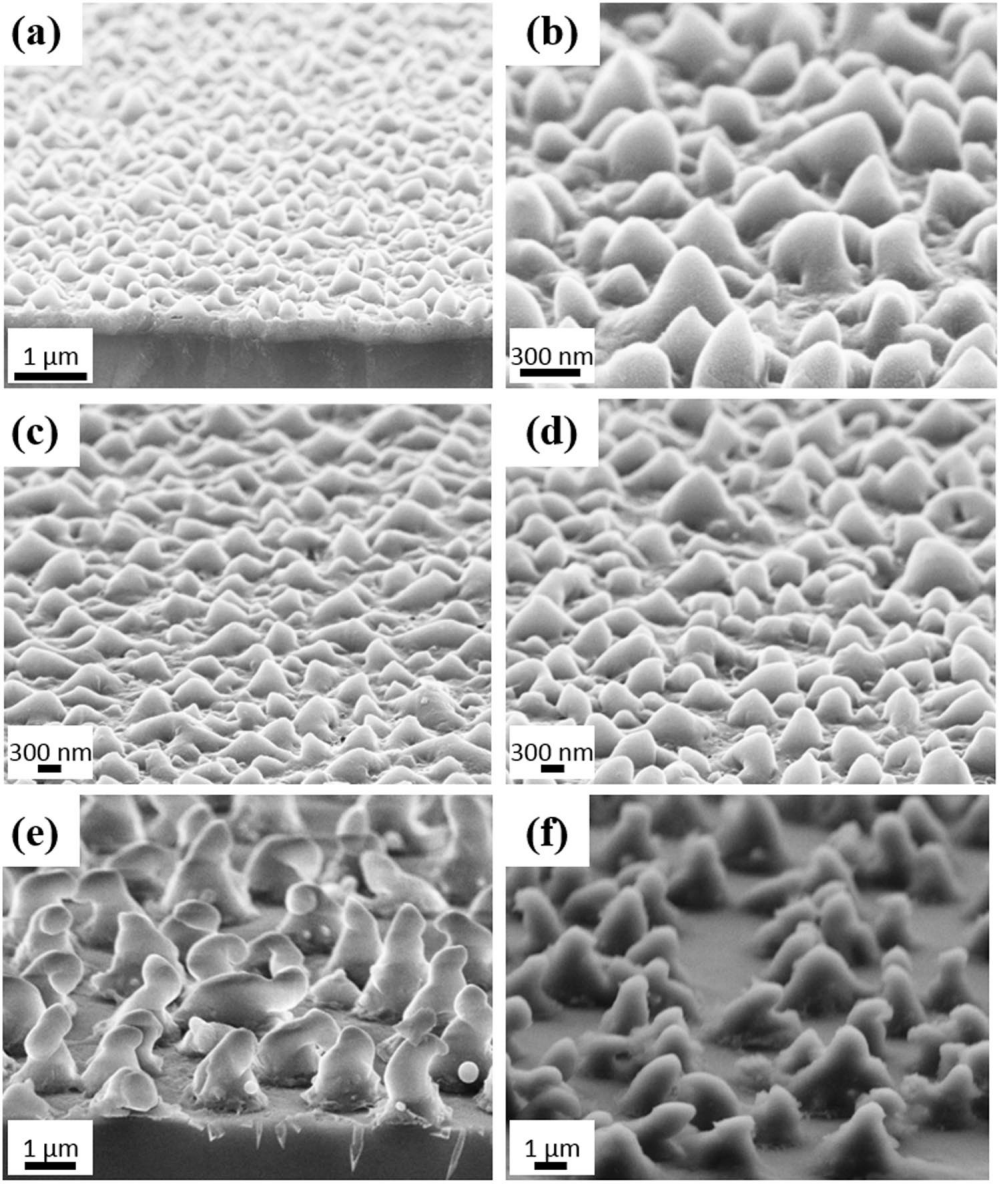
SEM images of NCs formed with increasing the energy density from 45 mJ/cm^2^ to (**a**,**b**) 260 mJ/cm^2^, (**c**) 300 mJ/cm^2^, (**d**) 350 mJ/cm^2^, (**e**) 390 mJ/cm^2^, and (**f**) 520 mJ/cm^2^. All images are taken with 20° tilt.

Arrays of random NCs were formed with different sizes depending on the value of the maximum energy. Silicon NCs were distributed throughout the irradiated annealed section. Figure 2(a) shows the NCs at the center of the section annealed with an E_max_ of 260 mJ/cm^2^. It could be seen that only the upper part of the deposited a-Si layer contributed in forming the NCs while leaving approximately a 350 nm thick a-Si layer underneath. This indicates that the NCs are formed by transferring the a-Si material from some parts to others not by etching any of the deposited a-Si.

**Figure 3:**
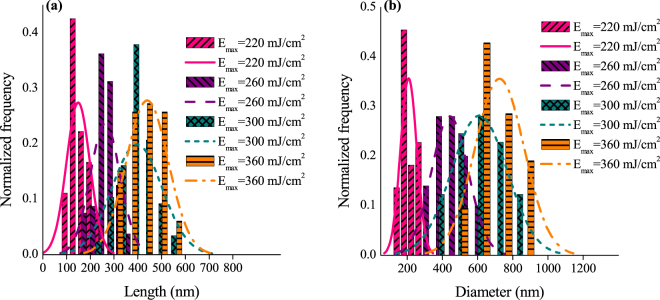
The histograms and the Gaussian distribution of (**a**) the length and (**b**) diameter for the samples with different E_max_.

Increasing the E_max_ increases the size of the NCs; the length and the diameter. Figure 2(b–f) show the NCs formed with E_max_ values of 260 mJ/cm^2^, 300 mJ/cm^2^, 350 mJ/cm^2^, 390 mJ/cm^2^ and 520 mJ/cm^2^, respectively. The length and diameter distribution for each case was measured. it could be seen from the SEM images that there is a large spread in the size of the NCs. To indicate the spread in the length and base diameter of the NCs, a histogram for the length and the diameter of each sample was calculated. From the histogram, the mean and the standard deviation were calculated and a Gaussian curve was plotted accordingly. The histograms along with the gaussian distribution for the samples with different E_max_ are shown in Fig. 3. In these measurements, the number of cones measured in each sample varied between 50 and 80 cones. The area chosen for measuring the cones’ size was varied from 5 to 10 µm^2^ according to the size of the cones.

**Figure 4:**
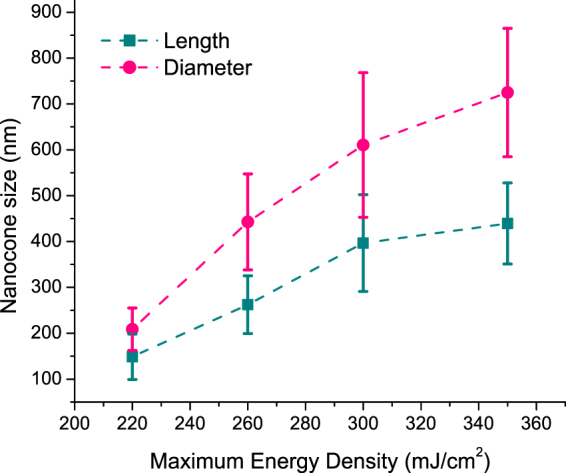
Effect of increasing the maximum energy density on the length (green cube line) and diameter (pink circle line) of the formed NCs.

Figure 3 shows accurately the spread in the length and the diameter of each measurement. To summarize the results and gain a better insight on the effect of the energy on changing the NC size, the mean of each measurement is plotted versus the energy for both the length and the diameter. The standard deviations are also indicated as shown in Fig. 4. NCs formed with an E_max_ of 220 mJ/cm^2^ had an average length of 148 nm while the base diameter is 209 nm. Increasing the E_max_ to 260 mJ/cm^2^ caused a rapid increase in the length (262 nm) and diameter (443 nm) Fig. 2(b). Further increase in E_max_ (300 mJ/cm^2^) increased the length to 397 nm and the diameter to 610 nm as shown in Fig. 2(c). For E_max_ equals 350 mJ/cm^2^, the length of the NCs reached 439 nm and the diameter 725 nm as shown in Fig. 2(d). It could be seen that NCs with lengths larger than the thickness of the deposited a-Si are obtained confirming that the transfer in material is the mechanism forming the NCs. Clearly, both the length and the base diameter of the NCs increase with increasing E_max_. The measured length of the NCs were done while considering the 20° tilt. The apparent length was divided by cos(20) to calculate the actual length without the tilt.

At 350 mJ/cm^2^, most of the deposited a-Si was already consumed in forming the NCs. Thus, any added energy resulted in forming irregular shapes as shown in Fig. 2(e) for E_max_ equals 390 mJ/cm^2^. Moreover, the layer started to be ablated with additional increase in energy as could be seen in the larger voids between the structures in Fig. 2(f) for E_max_ equals 520 mJ/cm^2^.

**Figure 5:**
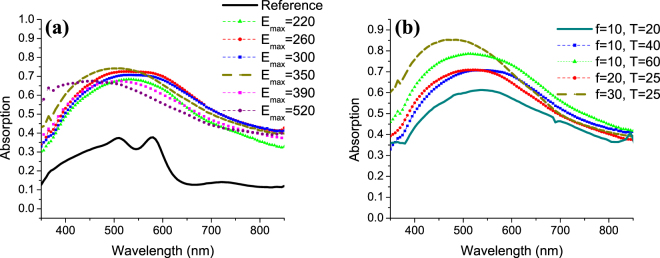
Absorption spectrum of a-Si irradiated by excimer laser with (**a**) different E_max_ while fixing f = 10 Hz and T = 40 s, and with (**b**) different frequencies and times while fixing E_max_ = 300 mJ/cm^2^.

To examine the optical properties of these nanostructured Silicon films, spectrophotometer analysis has been performed. A large, broadband increase in absorption has been detected for the NCs compared to the reference bare a-Si film without any nanostructuring. The as-deposited 370 nm a-Si has two absorption peaks at 520 nm and 580 nm as shown in Fig. 5(a). These peaks are attributed to the fabry perot resonances in the thin film. For wavelengths beyond 650 nm, the bare a-Si layer has a very low absorption of around 10%. At this small thickenss of a-Si, the losses at the long wavelength range become severe. Introducing the NCs on top of the a-Si resulted in a significant increase in absorption over the entire measured wavelength range as shown in Fig. 5(a). Increasing E_max_ from 220 mJ/cm^2^ to 260 mJ/cm^2^ resulted in an increase in absorption. Further increase in E_max_ resulted in increasing the absorption in the short wavelength range while a slight decrease was observed at the long wavelength range. It could be seen that at higher energies (E_max_ = 390 mJ/cm^2^ and 520 mJ/cm^2^), the absorption spectrum continued decreasing at the long wavelength range. The high absorption at the short wavelength observed for the E_max_ = 520 mJ/cm^2^ samples could be attributed to the larger length shapes obtained at this high energy. The decrease in absorption after E_max_ = 350 mJ/cm^2^ could be due to the larger voids between the cones and the depletion of silicon in these voids. The randomness of the produced NCs offers a variety of diameters that yields this broadband absorption enhancement.

**Figure 6:**
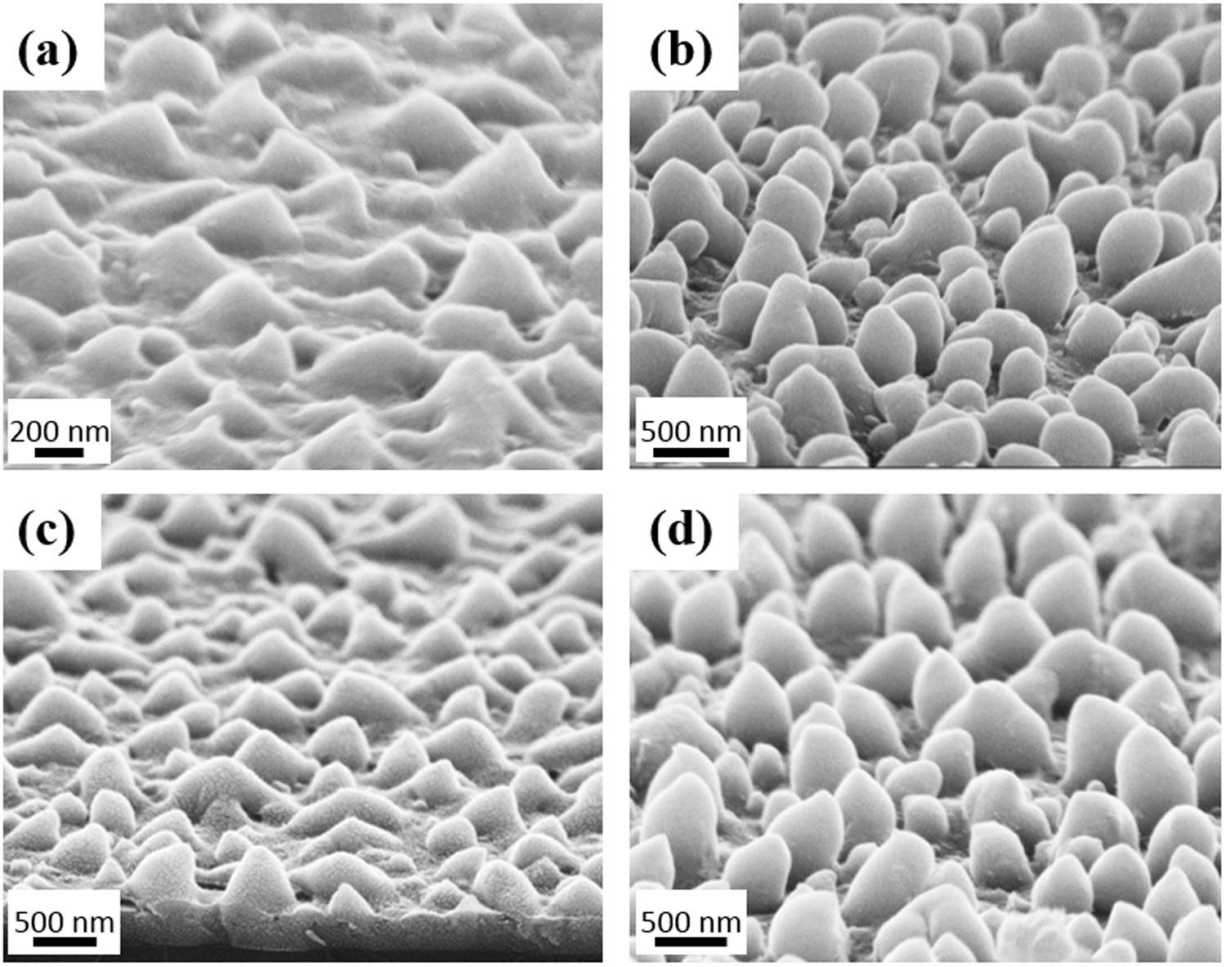
SEM images for NCs formed at fixed E_max_ = 300 mJ/cm^2^ and (**a**) T = 20 s, f = 10 Hz, (**b**) T = 60 s, f = 10 Hz, (**c**) T = 25 s, f = 20 Hz, and (**d**) T = 25 s, f = 30 Hz. All images are taken with 20° tilt.

In previous measurements, the energy was varied while keeping the time and frequency constant. Next, the effect of changing the time and frequency is studied while fixing E_max_ to 300 mJ/cm^2^. Decreasing the time in which the energy is gradually increased to 300 mJ/cm^2^, resulted in the formation of smaller NCs with an average length of 230 nm and a diameter of 290 nm as shown in Fig. 6(a). In addition, the density of the NCs is less compared to all other conditions with T = 40 s. This indicates that the NCs continue to form throughout the time of increasing the energy and not only at the beginning of the laser pulse irradiation. For these reasons, the decreased time condition showed the least absorption as shown in Fig. 5(b).

On the other hand, increasing the time to 60 s resulted in longer NCs with average length of 510 nm. However, the average diameter is only 415 nm. Hence, the structure in this case is different and has a length larger that the diameter. Figure 6(b) shows that this structure looks more like nanoparticles or extremely short nanowires.

Regarding the frequency variations, values were increased from the previous 10 Hz to 20 and 30 Hz. For higher frequency samples, the time was reduced to 25 s in order to prevent the formation of irregular shapes. Having a high frequency such as 30 Hz resulted in longer NCs, as shown in Fig. 6(d), with an average length of 545 nm and a diameter of 465 nm. Here, longer NCs are obtained without causing irregular shapes as in the case of increasing the energy density. These long NCs resulted in the highest absorption, especially in the short wavelength as shown in Fig. 5(b) for the NCs formed with 30 Hz in 25 s. The slightly reduced absorption in the long wavelength range may be attributed to the more rounded tip of the NCs compared to the sharp tip obtained with other conditions. It could be concluded that increasing any of the laser parameters (E_max,_ T or f) increases the length of the NCs.

**Figure 7:**
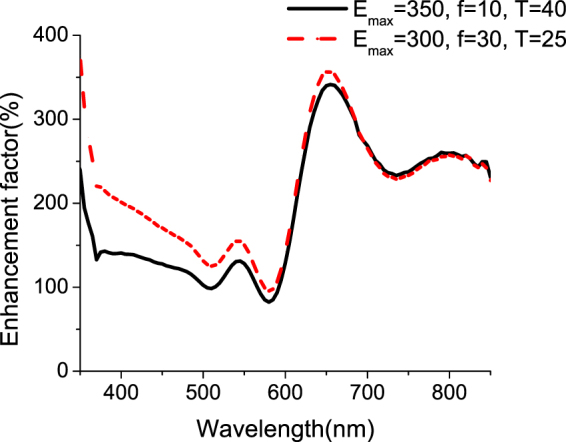
Enhancement factor for textured a-Si with (**a**) E_max_ = 350 mJ/cm^2^, f = 10 Hz, and T = 40 s, and (**b**) E_max_ = 300 mJ/cm^2^, f = 30 Hz, and T = 25 s.

The enhancement in absorption is calculated using the formula:

$$Enhancement\,factor({\rm{ \% }})=\frac{{\rm{A}}(\lambda )-{{\rm{A}}}_{{\rm{r}}{\rm{e}}{\rm{f}}}(\lambda )}{{{\rm{A}}}_{{\rm{r}}{\rm{e}}{\rm{f}}}(\lambda )}\times 100$$

where A(*λ*) is the absorption in the Silicon film after nanostructuring and A_ref_(*λ*) is the absorption in the bare a-Si film. More than 100% enhancement in absorption is calculated over the majority of the measured wavelength range except at a narrow band between 570 nm and 590 nm as shown in Fig. 7 for NCs fabricated with E_max_ = 350 mJ/cm^2^, f = 10 Hz and T = 40 s and NCs fabricated with E_max_ = 300 mJ/cm^2^, f = 30 Hz and T = 25 s. The NCs fabricated at 30 Hz show a higher enhancement factor in the short wavelength range. Up to 350% enhancement is obtained at the long wavelength range where the absorption in the bare a-Si layer was minimal due to the small thickness of the film.

The strong enhancement in light absorption is attributed to the reduced reflection of the textured Silicon layer compared to the flat one. Introducing a NC structure causes a gradual change in the refractive index resulting in supressing the reflection. In addition, this surface texturing increases light scattering in the a-Si under layer, resulting in increased light path length and thus increased absorption.

**Figure 8:**
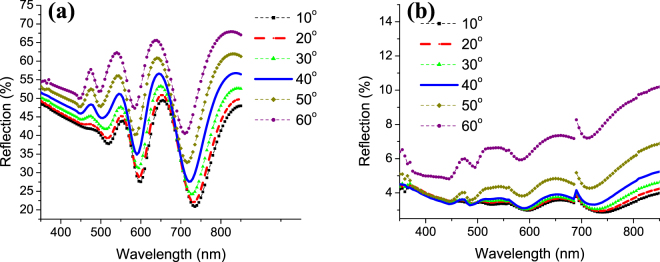
Reflection at angles from 10° to 60° for (**a**) bare a-Si and (**b**) textured a-Si with E_max_ = 350 mJ/cm^2^.

Another problem that arises for many Silicon solar cells is the angle dependent absorption. Away from the oblique light incidence, the reflection starts to increase causing a reduced absorption in the solar cell. For the bare 370 nm a-Si layer, the refection at angles from 10° to 60° is shown in Fig. 8(a). It could be seen that the two peaks found in the absorption correspond to two dips in the reflection due to the Fabry Perot resonance in the thin film. Except at these two dips, the reflection in the whole wavelength range is above 30% and increases with increasing the angle of incidence. The proposed structure with NCs shows a much reduced reflection not exceeding 5% in the whole wavelength range for angle of incidences up to 40°. At 50° and 60°, although the reflection slightly increases at longer wavelengths, it does not surpass 10% in all cases. This wide angle anti-reflective property is essential in the new generation of thin film Silicon solar cells and would play a significant role in the commercialization of such devices.

**Figure 9:**
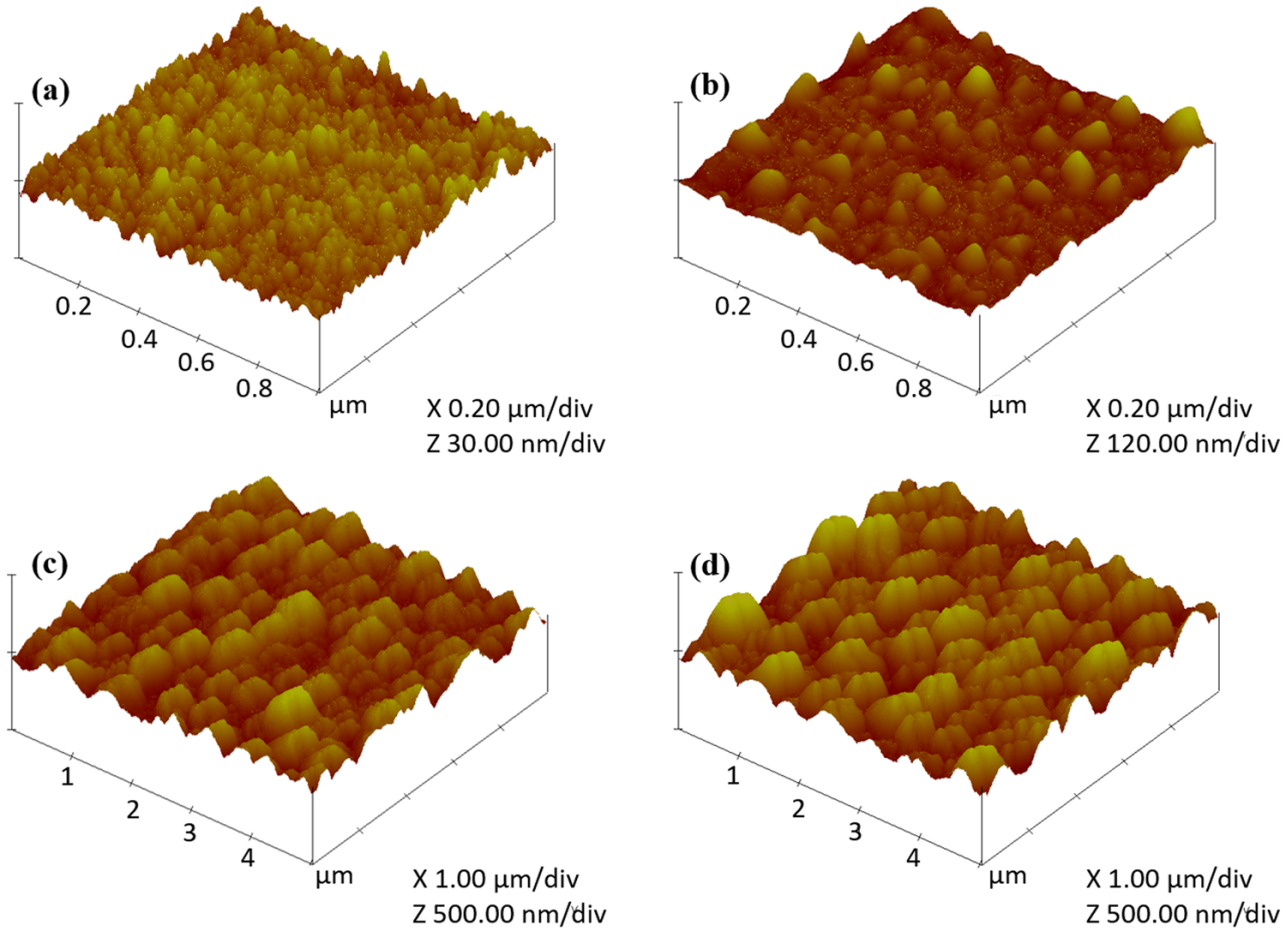
AFM images for each time interval for sample with E_max_ = 260 mJ/cm^2^. The energy density was increased (**a**) from 45 to 130 mJ/cm^2^ in 16 s, (**b**) from 45 to 175 mJ/cm^2^ in 24 seconds, (**c**) from 45 to 215 mJ/cm^2^ in 32 s. and (**d**) from 45 to 260 mJ/cm^2^ in 60 s.

To gain further insight on the phenomena occurring at each time interval while increasing the energy during laser irradiation, AFM is performed for 4 samples with gradually increasing the energy. The first sample had an energy density increasing from 45 to 130 mJ/cm^2^ in 16 s Fig. 9(a), the second one from 45 to 175 mJ/cm^2^ in 24 seconds Fig. 9(b), the third one from 45 to 215 mJ/cm^2^ in 32 s Fig. 9(c) and finally from 45 to 260 mJ/cm^2^ in 60 s Fig. 9(d). These images show the step-by-step progression of forming the nanocones in order to aid in analyzing the process of their formation by showing what happens at each time interval in these 60 s. In the first 16 s, small NCs (30 nm in length) are formed. Then, the length of NCs starts to increase in some areas in the sample reaching 120 nm as shown in Fig. 9(b). Additional increase in length (up to 500 nm) is observed as the energy density and the exposure time increase. Further increase in the energy density did not increase the length any further. However, more NCs were formed. It could be concluded from the AFM images that as the time pass while increasing the energy density, the size of the nanocones increase and the number of formed nanocones increases as well.

Due to the lower thermal conductivity of the silica substrate compared to Silicon, the absorbed heat due to the laser irradiation is mainly dissipated laterally^29^. Thus, the laser irradiation causes the surface of the deposited a-Si film to melt laterally. After laser induced melting, the Silicon surface starts to cool and re-solidifies. During re-solidification, the edges get more rapidly cooled than the center^29^. Hence, different densities arise in the same spot. Since the density of liquid Silicon (2.52 g/cm^3^) is larger than the density of solid amorphous Silicon (1.1 g/cm^3^), the liquid Silicon occupies a smaller volume than the solid a-Si^30,35^. Therefore, the volume of the Silicon increases during the solidification process.

**Figure 10:**
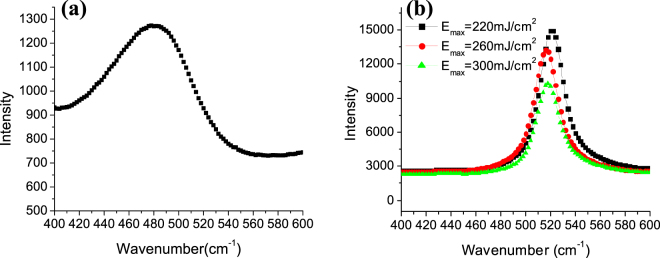
Raman spectrum for (**a**) as-deposited Silicon films and (**b**) Silicon film after laser irradiation.

Since the solidified material occupies a larger volume, it pushes the remaining liquid Silicon upward forming the NC. However, the NCs are formed as an array of several NCs within the same laser irradiated spot. This could be due to either the excitation of capillary waves or surface plasmon polaritons (SPP) waves^34,45^. The SPP waves are excited due to the excitation of large number of electrons in the conduction band during laser irradiation. Thus, the incident laser interferes with these surface waves causing periodic modulation of the laser light intensity, therefore, periodic structures are formed. In addition, during the cooling phase, a-Si material crystallizes while forming the NCs. This is confirmed by the Raman measurements shown in Fig. 10. While the as-deposited film shows a weak and broad peak located at ∼480 cm^−1^ corresponding to a typical a-Si peak Fig. 10(a), the laser annealed film exhibited a sharp peak centered around ∼520 cm^−1^, corresponding to a typical crystalline Silicon peak Fig. 10(b) ^44,45^.

## Conclusion

In summary, we report the fabrication of high, broadband, and wide angle absorption enhancement in Silicon nanocones on top of a thin film a-Si layer using excimer laser. The fabrication technique reported in this work is easily scalable, fast, and does not require any especial conditions or gases. The effect of changing the laser parameters such as energy density, time, and frequency on the morphology and the absorption are studied and a thorough comparison is made. Less than 6% reflection for angles of incidents up to 50° is reported. This work focused mainly on enhancing the optical efficiency of the solar cell. This study could be further extended by electrically connect the solar cell which could be done by depositing a p-type silicon on an n-type silicon nanocones or spin coating a conductive polymer to form the junction, and then depositing the metal electrode on top by evaporation. For the back electrode, the n-type silicon material could be deposited on a metal (e.g. Aluminum) instead of glass.

## Methods

### Cleaning and deposition

Glass slides are cleaned by immersing them in acetone then IPA for 15 minutes each, then rinsed with deionized water. They are then put in a piranha solution for 30 minutes and finally rinsed again with deionized water. The piranha solution is prepared by slowly adding H_2_O_2_ to Sulfuric acid with a ratio 1:4. Immediately after cleaning, the glass slides were put in the PECVD chamber for deposition. The cleaning and the deposition were done in a grade-100 clean room. The deposition was done using a SiH_4_ source and a He source with a 1:1 ratio. The He is used instead of hydrogen for faster and more uniform deposition. The table temperature is set to 250 °C and the chamber pressure to 1.5 Torr. The time was set to 20 minutes to obtain the desired thickness.

### Formation of nanocones

The deposited amorphous silicon was then irradiated by the excimer laser. A Lamda Physik KrF excimer laser is used (Compex205) with wavelength 248 nm. The pulse duration is 24 ns and the irradiated spot area is fixed to 23 mm^2^ using a double projection lens. Samples were mounted on a motorized computer controlled stage in a closed chamber. The chamber is maintained under a reduced pressure of 3.5 mbar during irradiation. The sample is adjusted in front of the laser beam then the laser is turned on for a specific amount of time. During laser irradiation, a protective eye glass was used. The laser frequency is adjusted prior to laser irradiation for each sample. The starting point of the laser energy density is adjusted prior to laser irradiation, and during laser irradiation, the laser energy density was manually increased to maximum energy density (E_max_). the time in which the laser energy density was gradually increased varied from sample to another but was typically few seconds. After laser irradiation, the chamber was vented to get back to normal room pressure and the sample was removed.

### Characterization

The SEM images were performed using ZEISS Leo field emission scanning electron microscopy (FESEM). All SEM images are taken for the cross section of the sample. Prior to SEM measurements, the samples were cut in the middle of the laser spot to be able to visualize their cross section. Then, they were sputtered with Au to make the surface conductive to be able to visualize it in the SEM. Au sputtering was done for 40 seconds using one target. After sputtering, the samples were placed inside the SEM chamber with their cross section facing the electron gun and titled with 20°. the chamber was evacuated to reach a base pressure of 1 × 10^−9^ mbar for gun vacuum and 1 × 10^−5^ mbar for chamber vacuum. In all measurements, the electron gun was adjusted in the middle of the laser irradiated spot so that the electron beam hit the sample in the middle. The contrast and brightness of each sample were adjusted to reach the optimum contrast for each image independently and the focus was adjusted accordingly. The scan rate for all images was adjusted so that each image was taken in 2 minutes. The InLens detector was used in the majority of the SEM images. In order to get clear images of the structures, the working distance was typically set between 1 and 3 mm and the acceleration voltage to 8 kV. The morphology analysis was examined using VEECO Dimension 3100 Atomic force microscopy (AFM). The tapping mode was used during the measurements with a sharpened DNP-S10 silicon nitride probe (tip radius = 10 nm and tip height = 2.5 um). For the first two samples, the AFM scan area was 1µmx1µm with a scan rate of 1.969 Hz. For the second two samples, the AFM scan area was 5µmx5µm with a scan rate of 1.001 Hz. For absorption and reflection measurements, a Perkin-Elmer Lambda 950 (UV-VIS NIR) spectrophotometer was used. The absorption is measured using a 150 mm integrated sphere unit (Lab Sphere). The wall of the integrated sphere unit as well as the reference are made of a spectralon material (a fluoropolymer that has a very high diffuse reflectance). The setting up of the experiment is done using a UV Winlab software application. The procedure for the measurements starts by first recording a baseline scan by doing 0% reflection and 100% reflection while there are no samples. The 0% reflection detects a dark signal, while blocking the beam, to measure the noise signal of the detector. The 100% reflection detect the reflection of the spectralon material as a reference measurement. The detector used in the visible range is a R955 photomultiplier tube (PMT) detector that is embedded in the internal walls of the integrated sphere unit. Figure S1 in the supplementary materials shows the beam directions inside the integrated sphere unit. After doing the baseline measurements, the sample is placed inside the integrated sphere unit. The PMT detector measures the percentage of the diffuse reflection from the sample compared to the reference (%R) and then measures the absorption according to the formula A = 2 − log_10_ (%R). Reflection at multiple angles were measured using the universal reflectance unit accessory which measure the specular reflection at variable angles. These measurements are done by first collecting the baseline measurements. Figure S2 in the supplementary materials shows the beam direction for collecting the baseline measurements inside the universal reflectance unit. For the baseline measurements, the input mirror rotates to direct the beam off the sample. The detector assembly moves vertically and with an angle to capture the reflected light according to the angle of incidence. The input mirror also moves horizontally to set the angle of incidence for the detector assembly. When the sample measurements are taken, the input mirror changes its angle to direct the beam to the sample as shown in Figure S3. The detector used for measurements in the UV-Vis range is a silicon detector. The Raman spectra were recorded at room temperature using ProRaman-L analyzer with the excitation wavelength of 532 nm, 66 mW laser diode and a linewidth 2.0 cm^−1^. The laser spot has a diameter of 0.7 mm and a Gaussian shape. The laser is fiber coupled to a 0.22 NA objective lens which is mounted adjacent to the detector. The sample is placed underneath the laser beam so that it is flat and perpendicular to the beam.

### Data and materials availability

All data needed to evaluate the conclusions in the paper are present in the paper. Additional data related to this paper may be requested from the author.

## Electronic supplementary material

Supplementary Information
