# Supplementary material for: One step fabrication of Silicon nanocones with wide-angle enhanced light absorption
Source: Sci Rep. 2018 Mar 5;8:4001. doi: 10.1038/s41598-018-22100-7 (PMC5838109; doi:10.1038/s41598-018-22100-7)
Supplement: Supplementary file 1 — Supplementary Information [file 41598_2018_22100_MOESM1_ESM.docx]

One step fabrication of Silicon nanocones with wide-angle enhanced light absorption supplementary materials

**Sara Magdi^1^, Joumana El-Rifai^2^, Mohamed A. Swillam^1, 2, a)^**

^1^Nanotechnology Program, American University in Cairo, AUC Avenue New Cairo 11835, Cairo, Egypt;

^2^Department of Physics, American University in Cairo, AUC Avenue New Cairo 11835, Cairo, Egypt.


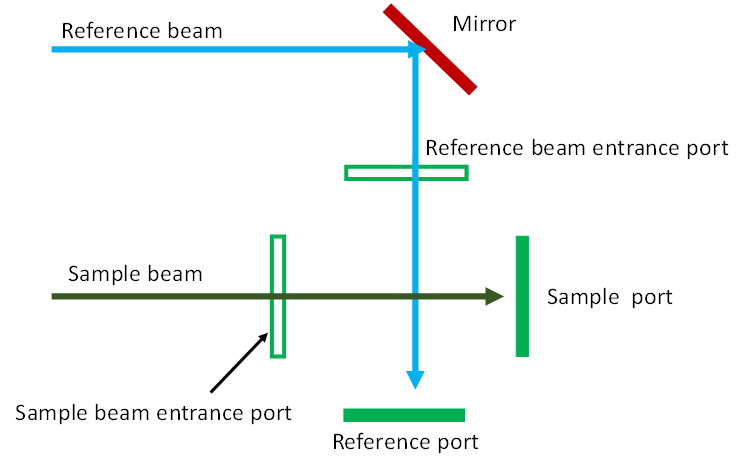


Figure S1:The beam directions inside the integrated sphere unit used to measure the absorption.

Figure S2: The beam directions for collecting the baseline measurements inside the universal reflectance unit.

Figure S3: The beam directions for collecting the sample measurements inside the universal reflectance unit.
